# Supplementary material for: Job Exposure Matrix for Chrysotile Asbestos Fibre in the Asbestos Cement Manufacturing (ACM) Industry in Zimbabwe
Source: Int J Environ Res Public Health. 2022 Feb 25;19(5):2680. doi: 10.3390/ijerph19052680 (PMC8909927; doi:10.3390/ijerph19052680)
Supplement: Supplementary file 1 [file ijerph-19-02680-s001.zip › ijerph-1576228-supplementary.pdf]

Supplementary Information

Job Exposure Matrix for Chrysotile Asbestos Fibre in the Asbestos Cement Manufacturing (ACM) Industry in Zimbabwe

Benjamin Mutetwa [1,*], Dingani Moyo [1,2,3] and Derk Brouwer [1]

[1] Faculty of Health Sciences School of Public Health, , University of the Witwatersrand, Johannesburg 2193, South Africa; moyod@iwayafrica.co.zw (D.M.); derk.brouwer@wits.ac.za (D.B.)
[2] Faculty of Medicine and Health Sciences, Midland State University, Gweru 054, Zimbabwe
[3] Department of Community Medicine, Faculty of Medicine, National University of Science and Technology, Bulawayo 029, Zimbabwe
[*] Correspondence: bjmtetwa@yahoo.com; Tel.: +263-773-429-838

Content

1. Table S1: Harare Factory Post Hoc Test: Multiple comparisons showing mean differences between time periods for various job categories that reaches significance (p<0.05).
2. Table S2: Bulawayo Factory Post Hoc Test: Multiple comparisons showing mean differences between time periods for various job categories that reaches significance (p<0.05).

Table S1: Harare factory post hoc test showing mean differences between time periods for various jobs

Job	Time-period		Mean Difference (I-J)	Std. Error	p-value	HSD 95% Confidence Interval	
						Lower Bound	Upper Bound
Saw cutting operator	1996-2000	2001-2008	.06543*	0.00297	0.000	0.06	0.07
		2009-2016	.11918*	0.00306	0.000	0.11	0.13
		2018-2020	.08893*	0.00402	0.000	0.08	0.10
	2001-2008	1996-2000	-.06543*	0.00297	0.000	-0.07	-0.06
		2009-2016	.05375*	0.00277	0.000	0.05	0.06
		2018-2020	.02350*	0.00380	0.000	0.01	0.03
	2009-2016	1996-2000	-.11918*	0.00306	0.000	-0.13	-0.11
		2001-2008	-.05375*	0.00277	0.000	-0.06	-0.05
		2018-2020	-.03025*	0.00387	0.000	-0.04	-0.02
	2018-2020	1996-2000	-.08893*	0.00402	0.000	-0.10	-0.08
		2001-2008	-.02350*	0.00380	0.000	-0.03	-0.01
		2009-2016	.03025*	0.00387	0.000	0.02	0.04
Moulded goods operator	1996-2000	2001-2008	-0.00415	0.00478	0.822	-0.02	0.01
		2009-2016	.05769*	0.00532	0.000	0.04	0.07
		2018-2020	0.00400	0.01300	0.990	-0.03	0.04
	2001-2008	1996-2000	0.00415	0.00478	0.822	-0.01	0.02
		2009-2016	.06184*	0.00494	0.000	0.05	0.07
		2018-2020	0.00815	0.01284	0.921	-0.03	0.04
	2009-2016	1996-2000	-.05769*	0.00532	0.000	-0.07	-0.04
		2001-2008	-.06184*	0.00494	0.000	-0.07	-0.05
		2018-2020	-.05369*	0.01305	0.000	-0.09	-0.02
	2018-2020	1996-2000	-0.00400	0.01300	0.990	-0.04	0.03
		2001-2008	-0.00815	0.01284	0.921	-0.04	0.03
		2009-2016	.05369*	0.01305	0.000	0.02	0.09
Kollergang operator	1996-2000	2001-2008	.01124*	0.00422	0.042	0.00	0.02
		2009-2016	.06414*	0.00445	0.000	0.05	0.08
		2018-2020	0.01358	0.00880	0.413	-0.01	0.04
	2001-2008	1996-2000	-.01124*	0.00422	0.042	-0.02	0.00
		2009-2016	.05290*	0.00411	0.000	0.04	0.06
		2018-2020	0.00235	0.00863	0.993	-0.02	0.02
	2009-2016	1996-2000	-.06414*	0.00445	0.000	-0.08	-0.05
		2001-2008	-.05290*	0.00411	0.000	-0.06	-0.04
		2018-2020	-.05056*	0.00874	0.000	-0.07	-0.03
	2018-2020	1996-2000	-0.01358	0.00880	0.413	-0.04	0.01
		2001-2008	-0.00235	0.00863	0.993	-0.02	0.02
		2009-2016	.05056*	0.00874	0.000	0.03	0.07
Ground hard waste operator	1996-2000	2001-2008	.02455*	0.00508	0.000	0.01	0.04
		2009-2016	.08472*	0.00510	0.000	0.07	0.10
		2018-2020	.03384*	0.01019	0.006	0.01	0.06
	2001-2008	1996-2000	-.02455*	0.00508	0.000	-0.04	-0.01
		2009-2016	.06017*	0.00513	0.000	0.05	0.07
		2018-2020	0.00929	0.01021	0.800	-0.02	0.04
	2009-2016	1996-2000	-.08472*	0.00510	0.000	-0.10	-0.07
		2001-2008	-.06017*	0.00513	0.000	-0.07	-0.05
		2018-2020	-.05089*	0.01022	0.000	-0.08	-0.02
	2018-2020	1996-2000	-.03384*	0.01019	0.006	-0.06	-0.01
		2001-2008	-0.00929	0.01021	0.800	-0.04	0.02
		2009-2016	.05089*	0.01022	0.000	0.02	0.08
Laundry room operator	1996-2000	2001-2008	0.00864	0.00461	0.243	0.00	0.02
		2009-2016	.08671*	0.00755	0.000	0.07	0.11
		2018-2020	.02761*	0.00775	0.003	0.01	0.05
	2001-2008	1996-2000	-0.00864	0.00461	0.243	-0.02	0.00
		2009-2016	.07807*	0.00712	0.000	0.06	0.10
		2018-2020	0.01897	0.00733	0.051	0.00	0.04
	2009-2016	1996-2000	-.08671*	0.00755	0.000	-0.11	-0.07
		2001-2008	-.07807*	0.00712	0.000	-0.10	-0.06
		2018-2020	-.05910*	0.00946	0.000	-0.08	-0.03
	2018-2020	1996-2000	-.02761*	0.00775	0.003	-0.05	-0.01
		2001-2008	-0.01897	0.00733	0.051	-0.04	0.00
		2009-2016	.05910*	0.00946	0.000	0.03	0.08

Table S2: Bulawayo factory post hoc test showing mean differences between time periods for various jobs

Job	Time-period		Mean Difference (I-J)	Std. Error	p-value	HSD 95% Confidence Interval	
						Lower Bound	Upper Bound
Saw cutting operator	1996-2000	2001-2008	.05125*	0.00401	0.000	0.0408	0.0617
		2009-2016	.11347*	0.00588	0.000	0.0982	0.1288
		2017-2019	.11380*	0.00496	0.000	0.1009	0.1267
	2001-2008	1996-2000	-.05125*	0.00401	0.000	-0.0617	-0.0408
		2009-2016	.06222*	0.00589	0.000	0.0469	0.0775
		2017-2019	.06255*	0.00497	0.000	0.0496	0.0755
	2009-2016	1996-2000	-.11347*	0.00588	0.000	-0.1288	-0.0982
		2001-2008	-.06222*	0.00589	0.000	-0.0775	-0.0469
		2017-2019	0.00033	0.00657	1.000	-0.0168	0.0174
	2017-2019	1996-2000	-.11380*	0.00496	0.000	-0.1267	-0.1009
		2001-2008	-.06255*	0.00497	0.000	-0.0755	-0.0496
		2009-2016	-0.00033	0.00657	1.000	-0.0174	0.0168
Kollergang operator	1996-2000	2001-2008	.02230*	0.00548	0.000	0.0080	0.0366
		2009-2016	.06915*	0.00577	0.000	0.0541	0.0842
		2017-2019	.07944*	0.00741	0.000	0.0601	0.0987
	2001-2008	1996-2000	-.02230*	0.00548	0.000	-0.0366	-0.0080
		2009-2016	.04685*	0.00556	0.000	0.0324	0.0613
		2017-2019	.05714*	0.00725	0.000	0.0382	0.0760
	2009-2016	1996-2000	-.06915*	0.00577	0.000	-0.0842	-0.0541
		2001-2008	-.04685*	0.00556	0.000	-0.0613	-0.0324
		2017-2019	0.01029	0.00748	0.516	-0.0092	0.0298
	2017-2019	1996-2000	-.07944*	0.00741	0.000	-0.0987	-0.0601
		2001-2008	-.05714*	0.00725	0.000	-0.0760	-0.0382
		2009-2016	-0.01029	0.00748	0.516	-0.0298	0.0092
Groundhard waste operator	1996-2000	2001-2008	0.02194	0.00993	0.130	-0.0042	0.0480
		2009-2016	.06227*	0.01445	0.000	0.0243	0.1003
		2017-2019	.07144*	0.01081	0.000	0.0430	0.0999
	2001-2008	1996-2000	-0.02194	0.00993	0.130	-0.0480	0.0042
		2009-2016	0.04033	0.01604	0.066	-0.0018	0.0825
		2017-2019	.04950*	0.01286	0.001	0.0157	0.0833
	2009-2016	1996-2000	-.06227*	0.01445	0.000	-0.1003	-0.0243
		2001-2008	-0.04033	0.01604	0.066	-0.0825	0.0018
		2017-2019	0.00917	0.01660	0.946	-0.0345	0.0528
	2017-2019	1996-2000	-.07144*	0.01081	0.000	-0.0999	-0.0430
		2001-2008	-.04950*	0.01286	0.001	-0.0833	-0.0157
		2009-2016	-0.00917	0.01660	0.946	-0.0528	0.0345
Pipe joints operator	1996-2000	2001-2008	.01948*	0.00594	0.008	0.0040	0.0350
		2009-2016	.07073*	0.01031	0.000	0.0438	0.0977
		2017-2019	.08095*	0.01330	0.000	0.0462	0.1157
	2001-2008	1996-2000	-.01948*	0.00594	0.008	-0.0350	-0.0040
		2009-2016	.05126*	0.01027	0.000	0.0244	0.0781
		2017-2019	.06148*	0.01327	0.000	0.0268	0.0961
	2009-2016	1996-2000	-.07073*	0.01031	0.000	-0.0977	-0.0438
		2001-2008	-.05126*	0.01027	0.000	-0.0781	-0.0244
		2017-2019	0.01022	0.01572	0.915	-0.0308	0.0513
	2017-2019	1996-2000	-.08095*	0.01330	0.000	-0.1157	-0.0462
		2001-2008	-.06148*	0.01327	0.000	-0.0961	-0.0268
		2009-2016	-0.01022	0.01572	0.915	-0.0513	0.0308
Multicutter operator	1996-2000	2001-2008	0.00338	0.00644	0.953	-0.0136	0.0204
		2009-2016	.05615*	0.01835	0.017	0.0077	0.1046
		2017-2019	.08115*	0.01835	0.000	0.0327	0.1296
	2001-2008	1996-2000	-0.00338	0.00644	0.953	-0.0204	0.0136
		2009-2016	.05278*	0.01817	0.026	0.0048	0.1008
		2017-2019	.07778*	0.01817	0.000	0.0298	0.1258
	2009-2016	1996-2000	-.05615*	0.01835	0.017	-0.1046	-0.0077
		2001-2008	-.05278*	0.01817	0.026	-0.1008	-0.0048
		2017-2019	0.02500	0.02501	0.750	-0.0410	0.0910
	2017-2019	1996-2000	-.08115*	0.01835	0.000	-0.1296	-0.0327
		2001-2008	-.07778*	0.01817	0.000	-0.1258	-0.0298
		2009-2016	-0.02500	0.02501	0.750	-0.0910	0.0410

HSD – Tukey Honest Significance Difference: The mean difference is significant at the 0.05 level.
